# Association of Human Gut Microbiota with Alzheimer’s Disease Pathogenesis: An Exploratory Clinical Study

**DOI:** 10.3390/brainsci16020242

**Published:** 2026-02-21

**Authors:** Tadashi Ohara, Yasuyuki Taki

**Affiliations:** 1 Department of Aging Research and Geriatric Medicine, Institute of Development, Aging and Cancer, Tohoku University, Miyagi 980-8575, Japan; 2 Smart Ageing International Research Center, Tohoku University, Miyagi 980-8575, Japan

**Keywords:** Alzheimer’s disease, brain–gut axis, human gut microbiota, pathogenesis, preclinical Alzheimer’s disease, MCI

## Abstract

**Background/Objectives:** The gut–brain axis and its role in neurological disorders have garnered increasing attention in recent years. However, studies assessing the association between microbiota and Alzheimer’s disease in Japanese cohorts are scarce. This study investigated the potential role of the brain–gut axis in Japanese patients with Alzheimer’s disease (AD), focusing specifically on the role of microbiota composition in AD pathogenesis. **Methods:** Ten patients with AD and 21 healthy adults across three different age groups were enrolled. Fecal microbiota composition was assessed using 16S rRNA gene sequencing. **Results:** We found that some bacterial species, such as *Ruminococcus inulinivorans* and *Ruminococcus torques*, were more abundant in the AD group, whereas others—including *Agathobacter rectalis*, *Bacteroides uniformis*, and *Clostridium butyricum*—were relatively more abundant in healthy adults. However, individual taxa differences did not reach statistical significance. **Conclusions:** Although our preliminary findings suggest no significant differences in fecal microbiota compositions between patients with AD and healthy individuals, they suggest that microbiota can identify a potential risk for AD development. Future research may help elucidate the bacterial species associated with AD pathogenesis, potentially enabling the use of microbiota composition as a screening tool to identify healthy individuals and those with AD or preclinical AD—an increasingly critical goal amid rising global dementia rates and the urgent need for preventive strategies.

## 1. Introduction

The increasing prevalence of dementia poses a significant societal challenge. Alzheimer’s disease (AD) accounts for approximately 70% of all dementia cases, progresses slowly, and is characterized by the accumulation of amyloid-β (Aβ) peptide in the brain and the hyperphosphorylation of tau protein, resulting in damage to brain nerve cells and their connections [1,2,3]. Among Aβ peptides, Aβ42 is highly hydrophobic and amyloidogenic, and elevated levels of Aβ42 are strongly associated with amyloid plaque formation [4,5,6]. Interestingly, studies have shown that germ-free mice lacking intestinal microbiota do not exhibit Aβ accumulation in the brain [7], which further supports the role of the gut–brain axis on neurodegenerative diseases.

Recently, it has been reported that transplantation of human feces from patients with AD into healthy rats causes AD in these rats [8] through changing the expression of caecal metabolites involved in the neurogenic and cognitive function, and that transplantation of feces from young mice into aged mice results in improved cognitive function and immunity [9]. Of particular interest is the intestinal bacterium *Clostridiales*, which produces an Aβ-like peptide with high homology to the human Aβ peptide, sharing a similar tertiary structure and Aβ42-like activity [10]. Experimental evidence from various mouse models indicates that dysbiosis may cause the breakdown of the intestinal barrier, thus allowing Aβ peptides, LPS and bacterial metabolites to enter the bloodstream, cross the blood–brain barrier, and accumulate in the brain, leading to dementia symptoms [10,11,12]. AD pathogenesis is thought to involve not only brain Aβ but also secondary factors such as inflammation, metabolic byproducts, and immune signaling, collectively shaping the disease state, and there is evidence suggesting that the gut is a significant source of Aβ in human brains [13,14]. Taken together, these findings indicate that intestinal bacteria contribute to Aβ accumulation in the brain and, consequently, to the pathophysiology of AD.

Although numerous studies have evaluated the gut microbiota in AD, e.g., [14,15], geographical variations in gut microbiota composition have been scarcely investigated. Given the role of diet on gut microbiota, it is pertinent to assume that geographical regions with specific dietary habits may lead to a characteristic microbiota composition. For example, unlike typical Western diets, Japanese diets include fermented foods, high fiber and seafood. However, the literature addressing the composition of gut microbiota in Japanese subjects both in health and disease remains scarce. Therefore, this study investigated the composition of gut microbiota in Japanese people, including healthy subjects and patients with AD. Specifically, we hypothesized that the human intestinal microbiota may contain certain bacteria that promote Aβ production (i.e., bacteria contributing to AD pathogenesis) and others that suppress Aβ accumulation (i.e., bacteria mitigating AD progression).

## 2. Materials and Methods

### 2.1. Participants

This exploratory study was conducted at Akirudai Hospital, with sample collection and testing taking place between 1 April 2023 and 20 May 2023. The study included all patients diagnosed with AD during that period who provided informed consent for participation. This group comprised 21 healthy adults aged 22–79 years (mean age: 40.6 years; 9 males and 12 females) and 10 patients with AD aged 78–92 years (mean age: 83.7 years; 3 males and 7 females). Healthy adults (HA) were further subdivided into three groups based on age: HA-1 (7 participants aged ≤30 years; 5 males and 2 females), HA-2 (4 participants aged 31–40 years; 3 males and 1 female), and HA-3 (10 participants aged ≥41 years; 4 males and 6 females). This allowed us to study microbiome composition across different ages, with the chosen age ranges distinguishing among young adults, adults and middle-aged and older participants. All participants provided written informed consent to take part in the study, including for sample collection and testing.

### 2.2. Inclusion and Exclusion Criteria

Healthy participants were outpatients without AD who consented to participate, had no known diseases, maintained a balanced diet, defined in accordance with the Japanese Food Guide Spinning Top [16], and were not taking medications that could influence their intestinal microbiota. Diet was assessed through a questionnaire, which was used only for participants’ selection and exclusion. A comprehensive medication history was collected, including prescription medications, over-the-counter drugs, and dietary supplements. Exclusion criteria for healthy participants included any antibiotic use within the prior 6 weeks, proton pump inhibitors or other acid-suppressing agents within the prior 4 weeks, probiotics or prebiotics within the prior 2 weeks, and other medications expected to markedly affect gut microbiota (e.g., immunosuppressants, glucocorticoids, bile acid sequestrants); a washout period was required if such medications had been used historically, with documentation of a minimum 2-week (short-acting) or longer (as appropriate) washout before sample collection. Both AD patients and healthy controls were excluded if they had comorbidities, a smoking history or dietary intolerances. Also, the groups had similar BMI (AD: 22.9 ± 1.10; HA-1: 21.86 ± 1.21; HA-2: 21.75 ± 0.96; HA-3: 22.1 ± 1.10).

For AD participants, inclusion criteria required a formal diagnosis of AD by a dementia specialist, alongside consent to participate. Exclusion criteria included (a) the use of antibiotics within the prior 6 weeks, (b) ongoing treatment with medications known to substantially affect intestinal microflora (e.g., PPIs, immunosuppressants, corticosteroids, or other agents listed above) within the specified washout periods, and (c) dietary intolerance.

### 2.3. Diagnosis of Alzheimer’s Disease

All AD participants were outpatients from the Neurology Department of Akirudai Hospital. A specialist with 30 years of experience in diagnosing dementia conducted the assessments, according to established criteria for AD diagnosis [17], including intellectual, cognitive, executive, and memory function tests, such as the Mini-Mental State Examination (MMSE), as well as neuropsychological evaluations. Imaging studies, including computed tomography (CT), magnetic resonance imaging (MRI), or amyloid positron emission tomography (PET), were performed to confirm brain and hippocampal atrophy and Aβ accumulation. All patients underwent all exams. Results of the MMSE are presented in Table 1.

### 2.4. Dietary and Medication Surveys

Dietary habits, which strongly influence intestinal microbiota composition, were assessed using a brief self-administered diet history questionnaire, aiming to exclude participants with atypical eating habits. Participants were also surveyed regarding the use of oral medications that could potentially impact gut microbiota composition [18].

Fecal sampling was performed after obtaining verbal and written informed consent from each donor, in accordance with ethical principles outlined in the Declaration of Helsinki. The study protocol was approved by the Ethics Review Committee of Akirudai Hospital (Approval Number: R517; Date of Approval: 23 February 2023).

### 2.5. Analysis of Fecal Microbiota

Stool samples were collected using commercially available stool collection kits (Techno Suruga Lab Co. Ltd., Shizuoka, Japan), which were distributed to the participants along with detailed collection instructions. Participants were instructed to store the samples at 4 °C until delivery to the hospital, and a transport container with an ice pack was provided. The bacterial flora in the collected fecal samples was analyzed using amplicon sequencing, heatmap hierarchical clustering, and diversity analysis (details in Text S1).

### 2.6. Amplicon Sequencing Analysis

DNA extracted from fecal samples was pretreated according to the method described by Takahashi et al. [19], and the crude DNA was purified using the GENE PREP STAR PI-480 DNA automatic separator (Kurashiki Boseki, Osaka, Japan). PCR amplification targeted the V3-V4 region of the bacterial 16S rRNA gene using the primer set 341f-R806 [20,21] that amplifies the V3-V4 region of the bacterial 16S rRNA gene according to the method described by Takahashi et al. [19]. An index sequence [22] unique to each sample was inserted into each primer at this step. Sequencing was performed using MiSeq ( MiSeq Software Updater v2.6, Illumina, San Diego, CA, USA) and MiSeq Reagent Kit v3 (600 cycles) (Illumina) for paired-end sequencing at 2 × 301 bp cycles.

### 2.7. Analysis of the Amplicon Sequencing Data

The primer sequences on paired-end sequencing reads were trimmed by Cutadapt version 1.18 with default settings [23]. Paired-end sequencing reads were merged using the fastq-join program with default settings [24]. Only merged reads with a quality score of ≥20 across more than 99% of the sequence were retained using FASTX-Toolkit (Fastx-toolkit version 0.0.13 requires libgtextutils-0.6) [25]. The chimeric sequences were deleted with usearch61 [26,27]. For taxonomic classification, the TechnoSuruga Lab Microbial Identification database (DB-BA) version 19.0 (TechnoSuruga Laboratory, Shizuoka, Japan) was used, as this is the most recent database available. Sequence with homology showing ≥97% identity to the DB-BA database was determined using the Metagenome@KIN analysis software, version 2.3 (World Fusion, Tokyo, Japan) [28,29].

For diversity analysis, the joined amplicon sequence reads were processed through QIIME2 ver 2020.6 [30]. Quality filtering and deletion of chimeric sequences were performed, after which representative sequences were created using DADA2 (Divisive Amplicon Denoising Algorithm 2) denoise-single ver 1.10.0 with default settings [31]. The samples were rarefied to a minimum of 19,180 sequences per sample, and alpha diversity indices (Chao1, Shannon, and Simpson) and beta diversity metric (weighted UniFrac, unweighted UniFrac, and Bray–Curtis distances) were calculated. The 2D-PCoA was also performed using the qiime2R version 0.99.13 [32] and tidyverse version 1.2.1 [33] libraries in R [34]. The statistical significance of chao1, Shannon, and Simpson indices among groups was assessed by the Kruskal–Wallis test. The statistical significance of similarity of bacterial communities among groups was assessed with the ANOSIM test.

### 2.8. Data Analysis Using the Statistical Analysis Software R

Using the species-level taxa estimated in the microbial identification database, bacterial species associated with AD and healthy participants were identified. Additionally, the composition of specific taxa, including genera *Bacteroides*, *phocaeie*, *Phocaeicola*, the family *Lachnospiraceae*, and the order *Clostridiales*, for which significant group differences had been found, was analyzed. R software, version 3.1.01 [34], was used to generate boxplots based on grouping information derived from the extracted species composition ratios.

Statistical comparisons were performed using the Mann–Whitney U test [35] using the vegan package version 2.5.7 [36,37] in R. The proportions of each bacterial species were evaluated for significant differences among the study groups. To control the false positive rate, *p*-values were corrected using the Bonferroni method [38]. Statistical significance was set at *p* < 0.05.

## 3. Results

**Figure 1:**
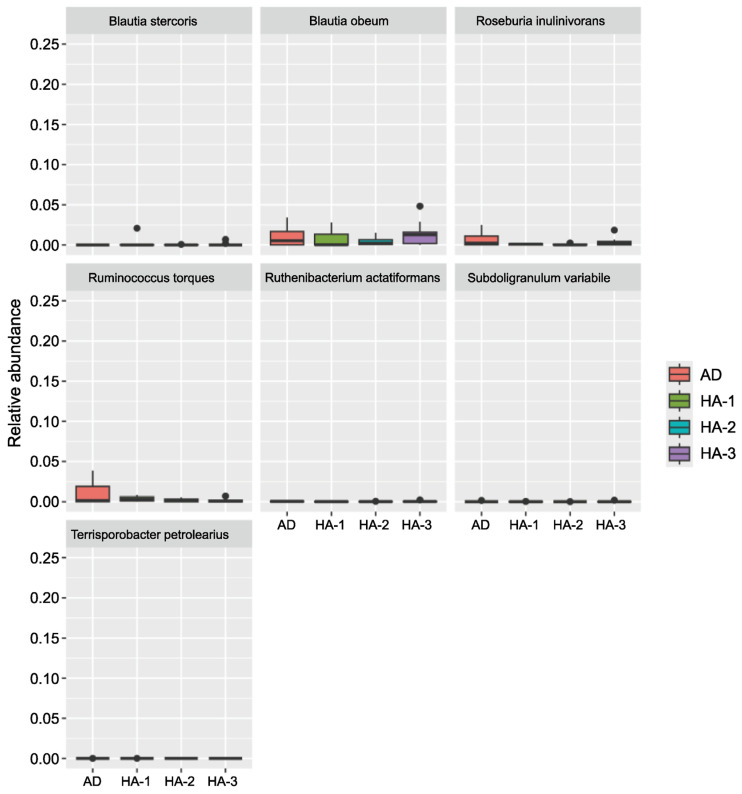
Bacterial species potentially associated with AD; all *p* > 0.05 (Mann–Whitney U test); AD: participants with Alzheimer’s disease (*n* = 10); HA-1: healthy participants aged ≤30 years (*n* = 7); HA-2: healthy participants aged 31–40 years (*n* = 4); HA-3: healthy participants aged ≥41 years (*n* = 10).

**Figure 2:**
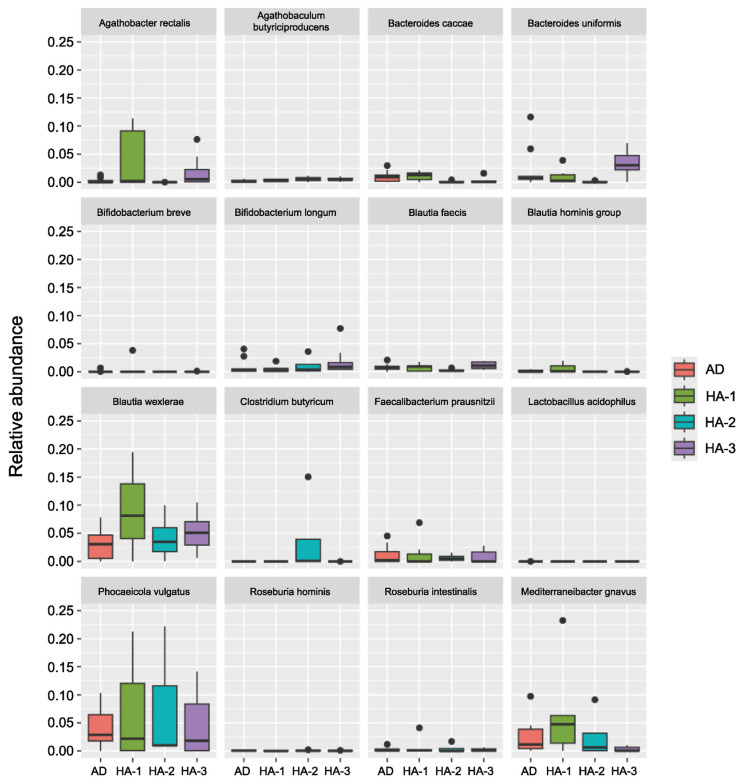
Bacterial species potentially associated with a non-AD state; all *p* > 0.05 (Mann–Whitney U test); AD: participants with Alzheimer’s disease (*n* = 10); HA-1: healthy participants aged ≤30 years (*n* = 7); HA-2: healthy participants aged 31–40 years (*n* = 4); HA-3: healthy participants aged ≥41 years (*n* = 10).

No significant differences were found in bacterial composition among the four groups (AD, HA-1, HA-2, and HA-3). Despite this, certain bacterial species were more commonly found in the AD group, whereas others appeared to predominate in healthy adults (*p* > 0.05). Composition variation was also found among the HA subgroups (HA-1, HA-2, and HA-3), which was also not significant (all *p* > 0.05), with exceptions for *Bacteroides uniformis* and *Mediterraneibacter gravus*, which were borderline significant between HA-2 and HA-3 and between AD and HA-3 (both *p* = 0.05), respectively (Figure 1 and Figure 2). Thus, *Ruminococcus torques* and *Roseburia inulinivorans* tended to predominate in individuals with AD, whereas *Agathobacter rectalis*, *Bacteroides uniformis*, *Blautia wexlerae*, and *Clostridium butyricum* predominated in healthy individuals.

The results of the heatmap hierarchical cluster analysis and diversity analysis are provided in Text S1, alongside Tables S1 and S2 and Figures S1–S8. Collectively, these analyses indicate that the diversity of the bacterial flora differed between the AD and healthy adult groups, with significant differences identified in the Bray–Curtis heatmap between AD and HA-2 (*p* = 0.035), AD and HA-3 (*p* = 0.003), and HA-1 and HA-3 (*p* = 0.019), and borderline significance between HA-2 and HA-3 (*p* = 0.06). No significant differences were found in the weighed or unweighted UniFrac heatmaps.

## 4. Discussion

In this exploratory study, we investigated the role of gut microbiota in the pathogenesis of AD within a Japanese cohort. Although our findings are exploratory, based on a small sample size, which could account for the lack of statistical significance, they suggest a predominance of several species of intestinal bacteria in the AD group and others in healthy adults. Recently, the gut–brain axis has garnered increasing attention for its role in neurological disorders [7], and prior research has demonstrated the influence of gut microbiota on AD [13]. Our findings potentially build on this evidence by providing preliminary data on this association in Japanese patients. Thus, our study may be a starting point for a better understanding of the effects of ethnicity on the gut–brain axis and its role in neurodegenerative diseases.

One of the species that appeared more abundant in the AD group was *R. inulinivorans*. This bacterium is a representative of the major Clostridial Cluster XIVa group, which is known for utilizing anaerobic polysaccharides to produce butyric acid during growth. *R. torques*, which was also more common in AD patients, is the most efficient degrader of mucin 2, a key component of colonic cell surface mucin. Increased intestinal permeability caused by *R. torques* activity has been implicated in AD pathogenesis [39]. *R. torques* also inhibits intestinal inflammation by producing secondary bile acids. Butyrate, a short-chain fatty acid produced by certain intestinal bacteria, is generally recognized for promoting mucin production, strengthening the mucosal layer and supporting intestinal barrier function. Additionally, butyrate increases the expression of trefoil factor family peptides, which contribute to the maintenance and repair of the intestinal mucosa and enhance the expression of tight junctions in colonic epithelial cells [40,41,42,43].

However, the effects of butyrate on the intestinal barrier are complex and concentration-dependent. Moderate concentrations of butyrate enhance intestinal barrier function, whereas excessively high concentrations can induce apoptosis in colonic epithelial cells, disrupt the intestinal barrier, and potentially contribute to colorectal cancer and other diseases. The precise role of butyrate in maintaining intestinal barrier integrity remains incompletely understood and warrants further investigation [44,45,46]. However, given our preliminary findings, it is pertinent to speculate that the higher quantity of *R. inulinivorans* found in AD patients could lead to a higher level of butyrate, thereby contributing to the pathogenesis of AD.

The treatment of AD dementia remains challenging because of concomitant neuronal damage and mitochondrial dysfunction caused by Aβ accumulation [47]. Lecanemab, a novel monoclonal antibody designed to degrade accumulated Aβ, has been developed as a treatment for AD [48]; another monoclonal antibody—donanemab—has also been evaluated in a phase 2 clinical trial, where it was found to improve cognition and performance in activities of daily living at 76 weeks [49], and has been recently approved for clinical use. Although these two therapies may slow the progression of AD dementia, they are not curative. Notably, no curative treatments for AD exist currently. Consequently, early screening for mild cognitive impairment (MCI) and AD is essential to prevent progression to AD dementia.

Previous studies have suggested the potential of screening for MCI and early AD by measuring plasma Aβ levels [50,51]. However, no effective screening methods for identifying individuals with preclinical AD have been established. In clinical cases of type 2 diabetes mellitus, intestinal bacteria play a key role in maintaining homeostasis until dysbiosis occurs [52], and some authors have suggested the potential to predict the onset of type 2 diabetes mellitus by analyzing the dynamics of intestinal bacteria [53]. Similarly, changes in intestinal microbiota composition and function have been observed to precede the onset of AD, suggesting that analyzing the intestinal microbiota could be a useful method to identify individuals at high risk of developing AD [54]. A recent review by Al Kuraishy et al. [55] highlights the potential value of calprotectin as a diagnostic and prognostic biomarker in Parkinson’s disease, based on its association with dysbiosis. Thus, further studies assessing its value as a biomarker in AD are warranted. These changes in intestinal microbiota are detectable prior to alterations in blood markers.

In humans, Aβ begins to accumulate in the brain at approximately 40 years of age, with the onset of MCI and AD occurring roughly 20 years later. During this intermediate period preceding AD onset, cognitive function appears normal [56]. Recent studies using experimental mouse models have demonstrated that intestinal bacteria produce Aβ, which enters the bloodstream, crosses the blood–brain barrier, and accumulates in brain tissue, ultimately leading to dementia [11,12]. Furthermore, emerging evidence suggests that the gut serves as a significant source of Aβ in human brains [13]. Besides accumulating in the brain, Aβ can be transported into the systemic circulation via a deranged blood–brain barrier and may accumulate in the heart, potentially contributing to heart failure [57]. Although the precise relationship between changes in intestinal microbiota and brain diseases remains unclear, regulation of dementia pathology via the brain–gut axis has become an increasingly prominent research area.

Many aspects of bacterial ecology and function remain poorly understood. Bacteria can acquire genetic material through crosstalk with their host or environment, exhibiting new functions, including those arising from horizontal gene transfer (HGT) events. A typical example of HGT is the acquisition of antibiotic resistance by bacteria. In multiple sclerosis (MS), an autoimmune demyelinating disease influenced by environmental factors and sharing some neurodegenerative pathways with AD, HGT has been reported to shape the pathogenicity of certain bacteria [58]. It is plausible that some bacterial genetic information mentioned above has been altered because of HGT events.

HGT analysis is not feasible with shotgun metagenomics, but long-read metagenomics enables precise analysis of bacterial chromosomes, making it a valuable tool for studying HGT. Long-read metagenomics can simultaneously detect and visualize HGT events in chromosomes across multiple bacterial species, facilitating detailed functional analysis of bacteria, including plasmid and bacteriophage interactions [59,60].

We hypothesize that the bacterial species potentially associated with AD dementia, as reported in this study, may possess Aβ-generating properties. To explore this hypothesis, we plan to analyze the functional genes of each intestinal bacterium comprehensively using long-read metagenomics, which will allow a more detailed understanding of the genetic mechanisms underlying their potential roles in AD pathogenesis.

We also investigated the functions of intestinal bacteria that were relatively more abundant in healthy adults. *C. butyricum* and *A. rectalis* are butyrate-producing bacteria, contributing to gut barrier integrity and anti-inflammatory effects. *B. wexlerae* produces metabolites such as ornithine, acetylcholine, and S-adenosylmethionine, which inhibit fat accumulation and work cooperatively with other intestinal bacteria to improve the intestinal environment, potentially preventing or mitigating obesity and diabetes.

Although various bacterial species have demonstrated anti-inflammatory, anti-obesity, and glycolytic effects, only *B. breve* and *A. butyriciproducens* have been reported to prevent dementia. We hypothesize that the bacterial species mentioned above may also exhibit anti-dementia or Aβ-decomposing effects. To investigate this hypothesis, we plan to use long-read sequence metagenomics for comprehensive intestinal microbiota gene analysis.

Analyzing the functional roles of intestinal bacteria must account for the effects of their metabolites. While genome analysis provides insights into bacterial genetic capabilities, metabolome analysis is equally essential for understanding bacterial functions.

Factors influencing intestinal microbiota composition include sex, dietary habits, and medications [61,62]. Of these, dietary habits and oral medications are particularly significant. Multivariate analyses incorporating metadata as confounding factors have revealed that drugs such as gastrointestinal medications, anti-diabetic drugs, antibiotics, antithrombotic agents, cardiovascular and brain disease treatments, and anticancer drugs, in that order, have the most substantial effects on gut microbiota [60]. Specifically, proton pump inhibitors, potassium-channel acid blockers, osmotic laxatives, amino acids, and bile acid inhibitors have strong effects among gastrointestinal drugs, while alpha-glucosidase inhibitors have notable effects among anti-diabetic medications.

Nagata et al. [63] also reported that multi-drug administration increases the number of opportunistic pathogens among commensal bacteria and decreases species producing short-chain fatty acids (SCFAs), such as butyric and acetic acid. In our study, although some participants were taking antihypertensive drugs, none were taking medications known to strongly affect gut microbiota or multiple drugs simultaneously. Additionally, none of the participants exhibited dietary deviations.

This exploratory study provides a first insight into the composition of gut microbiota in Japanese subjects. Further longitudinal studies are warranted to better understand how this composition changes over time. We identified bacterial species that tended to be relatively more abundant in the AD group and others that were relatively more abundant in the healthy adults. If our preliminary findings could be confirmed by larger studies, this would suggest the existence of an intermediate group between AD patients and healthy controls. This intermediate group, referred to as the “AD reserve group,” might represent individuals at heightened risk of developing dementia. However, this hypothesis requires validation in future large-scale clinical trials. Based on these findings, further studies on preventive strategies such as diet manipulations and the use of probiotics are needed, ultimately leading to the implementation of AD prevention projects. Currently, we are considering an AD prevention project using the bacterial species identified in this study.

Furthermore, the next step will be to consider the use of artificial intelligence to analyze the ratio (P/S) of bacterial species associated with AD onset (P) to those associated with AD suppression (S). If validated, the hypothesis that the P/S ratio increases in stages from healthy to preclinical AD to MCI/AD could enable rigorous diagnosis of the preclinical AD group. This approach has the potential to become a groundbreaking diagnostic tool for preclinical AD screening. Moreover, improving the composition of the intestinal microbiota through dementia-protective foods or the transplantation of AD-suppressing bacteria into individuals in the preclinical AD group could serve as a preventive strategy against AD [64,65,66,67]. In this context, larger studies are warranted, including an age- and sex-representative population, to validate the preclinical AD status.

Additionally, examining the dynamics of bacteria associated with AD and non-AD states before and after the ingestion of anti-dementia foods and food ingredients could facilitate rigorous evaluation of their cognitive effects. Such research could also enable the development of novel anti-dementia foods and ingredients, further contributing to AD prevention strategies.

There are several limitations to the current study. First, as this was a single-center study involving only 31 participants, the statistical power and the generalizability of the study were limited. Larger, multi-center studies with prospectively determined sample sizes based on a formal power analysis are warranted to validate the findings. Given the sample size, this study is hypothesis-generating rather than confirmatory. Second, the average age of the healthy adult population (35.3 years) was much lower than that of the AD population, which is particularly relevant given that the gut microbiome composition changes gradually with age [68]. To address this limitation, we included three groups of healthy adults with different age ranges and analyzed potential age-related differences in microbiota composition. However, the small sample size likely precluded the detection of statistically significant differences, making our study hypothesis-generating and not confirmatory, thus emphasizing the need for larger studies. Third, while our findings suggest potential associations between specific bacterial species and AD, they do not establish causation. Future longitudinal or interventional studies are needed to assess temporal changes and causality; employ deeper sequencing approaches (e.g., shotgun metagenomics, metatranscriptomics) to achieve higher taxonomic resolution and functional insights; integrate multi-omics (metabolomics) to link taxa with metabolites and host responses.

## 5. Conclusions

Analysis of fecal bacteria from healthy individuals and patients with AD identified bacterial species in the human intestinal microbiota that may be differentially associated with AD versus non-AD states in a Japanese cohort. These findings align with patterns observed in other ethnicities, offering promising insights into the potential for gut microbiota as a modifiable factor in AD prevention and possibly offering a noninvasive avenue for risk stratification and prevention in this population. However, the exploratory nature of the study makes these findings hypothesis-generating rather than confirmatory, given the small sample size, age-unmatched groups, and impossibility to address inter-individual differences. This underscores the need for large-scale clinical trials and detailed functional analyses of these bacterial species using advanced metagenomic techniques to validate these observations.

Furthermore, given the potential for gut microbiota reconstitution through supplementation [69], long-term interventional studies are needed to assess whether modifying the gut microbiome can influence the risk of developing AD, particularly in at-risk groups. Such studies would contribute significantly to the development of novel prevention strategies and interventions for AD.

## Figures and Tables

**Table 1:** Results from the Mini-Mental State Examination in Alzheimer’s Disease patients (*n* = 10).

Patient	Gender	Total Score
1	Male	18
2	Male	17
3	Male	20
4	Female	15
5	Female	15
6	Female	16
7	Female	18
8	Female	17
9	Female	15
10	Female	15

## Data Availability

The data supporting the findings of this study are available within the article and its Supplementary Materials.

## Supplementary Materials

The following supporting information can be downloaded at: https://www.mdpi.com/article/10.3390/brainsci16020242/s1. Text S1: Methods and Results for the heatmap hierarchical cluster analysis and diversity analysis; Table S1: Results of pairwise comparisons for alpha diversity; Table S2: Results of pairwise comparisons for beta diversity; Figure S1: Heatmap hierarchical cluster analysis results at the species level; Figure S2: Heatmap hierarchical cluster analysis results at the genus level; Figure S3: Heatmap hierarchical cluster analysis results at the family level; Figure S4: Heatmap hierarchical cluster analysis results at the order level; Figure S5: Heatmap hierarchical cluster analysis results at the class level; Figure S6: Heatmap hierarchical cluster analysis results at the phylum level; Figure S7: Alpha diversity rarefaction plots for the (A) Chao1, (B) Shannon, and (C) Simpson indices at a sequencing depth of 19,049 sequences per sample. AD: Alzheimer’s disease; HA: healthy adults; Figure S8: Three-dimensional principal component analysis plots showing (A) Bray–Curtis distances, (B) unweighted UniFrac distances, and (C) weighted UniFrac distances visualized using the emperor plot tool function in QIIME2. The red dots represent the AD group, the blue dots the HA-1 group, the orange dots the HA-2 group, and the green dots the HA-3 group [70,71,72,73].

## Abbreviations

The following abbreviations are used in this manuscript:

AD	Alzheimer’s disease
Aβ	amyloid-β
AI	artificial intelligence
APP/PSS1	amyloid precursor protein/phosphatidylserine synthase-1
CT	computed tomography
HGT	horizontal gene transfer
HA	healthy adults
IBS	irritable bowel syndrome
MCI	mild cognitive impairment
MRI	magnetic resonance imaging
MS	multiple sclerosis
PET	positron emission tomography
SCFAs	short-chain fatty acids
