# Supplementary material for: Association of Human Gut Microbiota with Alzheimer’s Disease Pathogenesis: An Exploratory Clinical Study
Source: Brain Sci. 2026 Feb 21;16(2):242. doi: 10.3390/brainsci16020242 (PMC12938429; doi:10.3390/brainsci16020242)
Supplement: Supplementary file 1 [file brainsci-16-00242-s001.zip › Supplementary_tables-edited.pdf]

Supplementary Tables

Table S1. Results of pairwise comparisons for alpha diversity

Group 1	Group 2	Alpha diversity index	H	p-value	q-value
AD	HA-1	chao1	3,6259	0,0569	0,1707
AD	HA-2	chao1	1,2800	0,2579	0,3868
AD	HA-3	chao1	0,3657	0,5454	0,6544
HA-1	HA-2	chao1	0,0807	0,7763	0,7763
HA-1	HA-3	chao1	5,0381	0,0248	0,1488
HA-2	HA-3	chao1	2,4200	0,1198	0,2396
AD	HA-1	shannon	3,8095	0,0510	0,1320
AD	HA-2	shannon	1,6200	0,2031	0,3046
AD	HA-3	shannon	0,1429	0,7055	0,7055
HA-1	HA-2	shannon	0,3214	0,5708	0,6849
HA-1	HA-3	shannon	3,4381	0,0637	0,1320
HA-2	HA-3	shannon	3,3800	0,0660	0,1320
AD	HA-1	simpson	3,0857	0,0790	0,1776
AD	HA-2	simpson	2,8800	0,0897	0,1776
AD	HA-3	simpson	0,0914	0,7624	0,7624
HA-1	HA-2	simpson	0,5714	0,4497	0,5396
HA-1	HA-3	simpson	2,4381	0,1184	0,1776
HA-2	HA-3	simpson	2,8800	0,0897	0,1776

Table Notes
AD: Alzheimer's disease; **HA**: healthy adults; **HA-1**: adults aged ≤30 years (5 males and 2 females); **HA-2**: adults aged 31–40 years (3 males and 1 female); **HA-3**: adults aged ≥41 years (4 males and 6 females); **a**: Benjamini and Hochberg corrected p-values (q-values) for Kruskal–Wallis tests.
*Statistically significant differences based on a p-value threshold of < 0.05.

Table S2. Results of pairwise comparisons for beta diversity

Group 1	Group 2	Sample size	Permutations	Beta Diversity Metrics	R	p-value	q-value
AD	HA-1	17	999	unweighted_unifrac	0,178	0,041	0,1280
AD	HA-2	14	999	unweighted_unifrac	0,187	0,151	0,1812
AD	HA-3	20	999	unweighted_unifrac	0,103	0,057	0,1280
HA-1	HA-2	11	999	unweighted_unifrac	-0,026	0,507	0,5070
HA-1	HA-3	17	999	unweighted_unifrac	0,149	0,064	0,1280
HA-2	HA-3	14	999	unweighted_unifrac	0,239	0,086	0,1290
AD	HA-1	17	999	weighted_unifrac	0,120	0,101	0,2300
AD	HA-2	14	999	weighted_unifrac	0,412	0,022	0,1320
AD	HA-3	20	999	weighted_unifrac	0,016	0,314	0,3140
HA-1	HA-2	11	999	weighted_unifrac	0,177	0,115	0,2300
HA-1	HA-3	17	999	weighted_unifrac	0,035	0,279	0,3140
HA-2	HA-3	14	999	weighted_unifrac	0,182	0,161	0,2415
AD	HA-1	17	999	bray_curtis	0,114	0,118	0,1416
AD	HA-2	14	999	bray_curtis	0,359	0,035	0,0700
AD	HA-3	20	999	bray_curtis	0,198	0,003	0,0180
HA-1	HA-2	11	999	bray_curtis	0,130	0,203	0,2030
HA-1	HA-3	17	999	bray_curtis	0,204	0,019	0,0570
HA-2	HA-3	14	999	bray_curtis	0,313	0,060	0,0900

Table Notes

AD: Alzheimer's disease; **HA**: healthy adults; **HA-1**: adults aged ≤30 years (5 males and 2 females); **HA-2**: adults aged 31–40 years (3 males and 1 female); **HA-3**: adults aged ≥41 years (4 males and 6 females); **a**: Benjamini and Hochberg corrected p-values (q-values) for ANOSIM tests. *Statistically significant differences based on a p-value threshold of < 0.05.
