# Supplementary material for: Association of Human Gut Microbiota with Alzheimer’s Disease Pathogenesis: An Exploratory Clinical Study
Source: Brain Sci. 2026 Feb 21;16(2):242. doi: 10.3390/brainsci16020242 (PMC12938429; doi:10.3390/brainsci16020242)
Supplement: Supplementary file 1 [file brainsci-16-00242-s001.zip › Text_S1-edited.pdf]

Text S1. Methods and Results for the Heatmap Hierarchical Cluster Analysis and Diversity Analysis

---

Methods

Heatmap Hierarchical Cluster Analysis

A heatmap hierarchical cluster analysis was conducted using the R packages gplots version 3.1.1 and a map version 0.8.18. Taxonomic data for the analysis were obtained from the NGS-DB-BA 16.0 database [34, 70, 71].

Diversity Analysis

The primer sequences from paired-end sequencing reads were trimmed using cutadapt software version 1.18 with default settings [23]. Paired-end sequencing reads were merged using the fastq-join program version 1.3.1 with default settings [24]. The joined amplicon sequence reads underwent further processing using QIIME2 software version 2020.6 [30]. Quality filtering and chimeric sequence removal were performed, and representative sequences were generated using the DADA2 (Divisive Amplicon Denoising Algorithm 2) denoise-single version 1.10.0 with default settings [31].
Taxonomy assignment of representative sequences was completed using the Greengenes Database version 13.8 138 [72], with a Naive Bayes classifier trained for the dataset. Samples were rarefied to a minimum of 19,049 sequences per sample to ensure uniform sequencing depth. Alpha diversity indices (Chao1, Shannon, and Simpson) and beta diversity metrics (weighted UniFrac, unweighted UniFrac, and Bray–Curtis distances) were calculated.
A 2D Principal Coordinate Analysis (PCoA) was performed using the qiime2R version 0.99.13 [32] and tidyverse version 1.2.1 [33] libraries in R [73]. Three-dimensional ordination plots were visualized using the emperor plot function in QIIME2. Statistical comparisons of alpha diversity indices (Chao1, Shannon, and Simpson) across groups—Alzheimer's disease (AD), adults aged ≤30 years (HA-1), adults aged 31–40 years (HA-2), and adults aged ≥41 years (HA-3)—were conducted using Kruskal–Wallis tests with Benjamini and Hochberg corrected p-values (q-values). Beta diversity similarity among groups was assessed using ANOSIM tests with Benjamini and Hochberg corrected p-values (q-values).
For alpha and beta diversity indices, 13 of the 23 cases were included in the analysis.

Results

Heatmap Hierarchical Cluster Analysis

The results of the heatmap hierarchical cluster analysis are presented in S1 Figure (species), S2 Figure (genus), S3 Figure (family), S4 Figure (order), S5 Figure (class), and S6 Figure (phylum). These heatmaps illustrate the clustering patterns of bacterial taxa at different taxonomic levels, highlighting variations between the AD and HA groups.

Diversity Analysis

The alpha diversity rarefaction plots for the Chao1, Shannon, and Simpson indices are shown in S7 Figure. Marginally significant differences were observed for the Chao1 index between the AD and HA-1 groups (Kruskal–Wallis test, Benjamini and Hochberg corrected p-values [q-values]; H = 3,6259, p = 0.0569, q = 0.1707, S1 Table).

A two-dimensional screenshot of the three-dimensional principal component analysis (PCA) plots for beta diversity analyses using Bray–Curtis, unweighted UniFrac, and weighted UniFrac metrics is shown in S8 Figure. The pairwise ANOSIM results (S2 Table) revealed significant differences between the AD and HA-1 groups for the Unweighted Unifrac beta diversity metrics (R = 0.178, p = 0.041, q = 0.1280). Significant differences were also found between AD and HA-2 groups, both for Weighted Unifrac and Bray–Curtis beta diversity metrics (Weighted Unifrac: R = 0.187, p = 0.022, q = 0.1320; Bray–Curtis: R = 0.359, p = 0.035, q = 0.0700), between AD and HA-3 for Bray–Curtis (R = 0.198, p = 0.003, q = 0.0180), and between HA-1 and HA-2 for Bray–Curtis (R = 0.204, p = 0.019, q = 0.0570). Moreover, a marginally significant difference was found between AD and HA-3 for Unweighted Unifrac beta diversity metrics (R = 0.103, p = 0.0570, q = 0.1280).

These findings collectively suggest that the diversity of intestinal flora differed significantly between the AD and HA groups, particularly in the HA-2 group. Further analyses are required to determine the biological implications of these differences.
