# Supplementary figures and images for: Association of Human Gut Microbiota with Alzheimer’s Disease Pathogenesis: An Exploratory Clinical Study
Source: Brain Sci. 2026 Feb 21;16(2):242. doi: 10.3390/brainsci16020242 (PMC12938429; doi:10.3390/brainsci16020242)

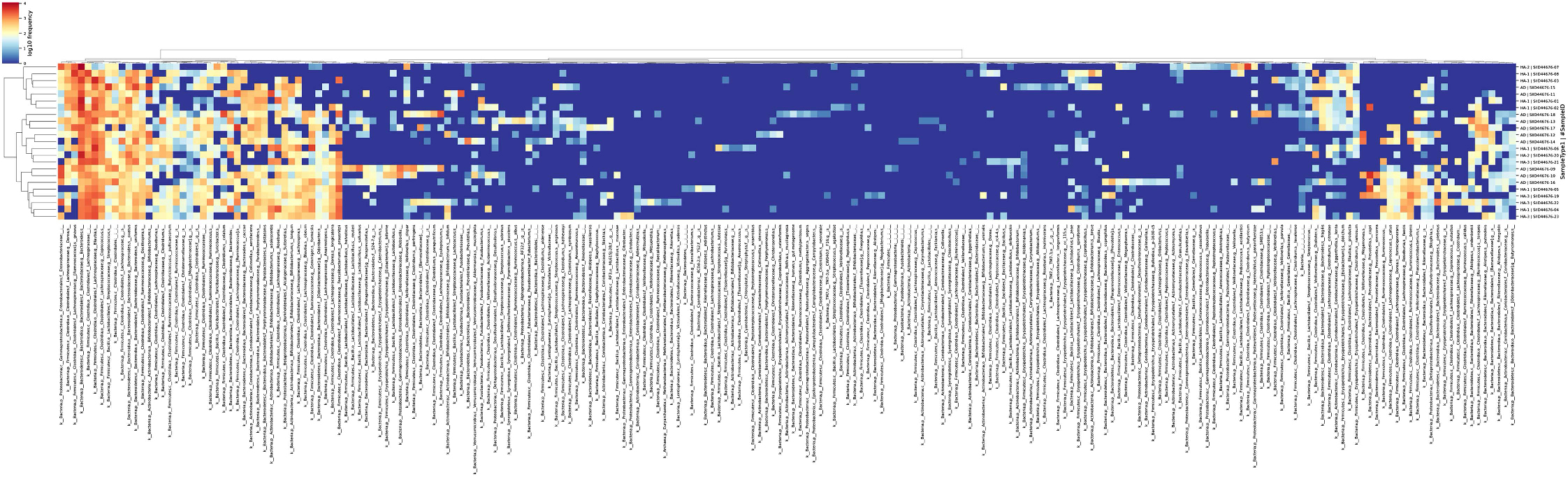

Supplement: Supplementary file 1 [file brainsci-16-00242-s001.zip › Fig S1.jpg]

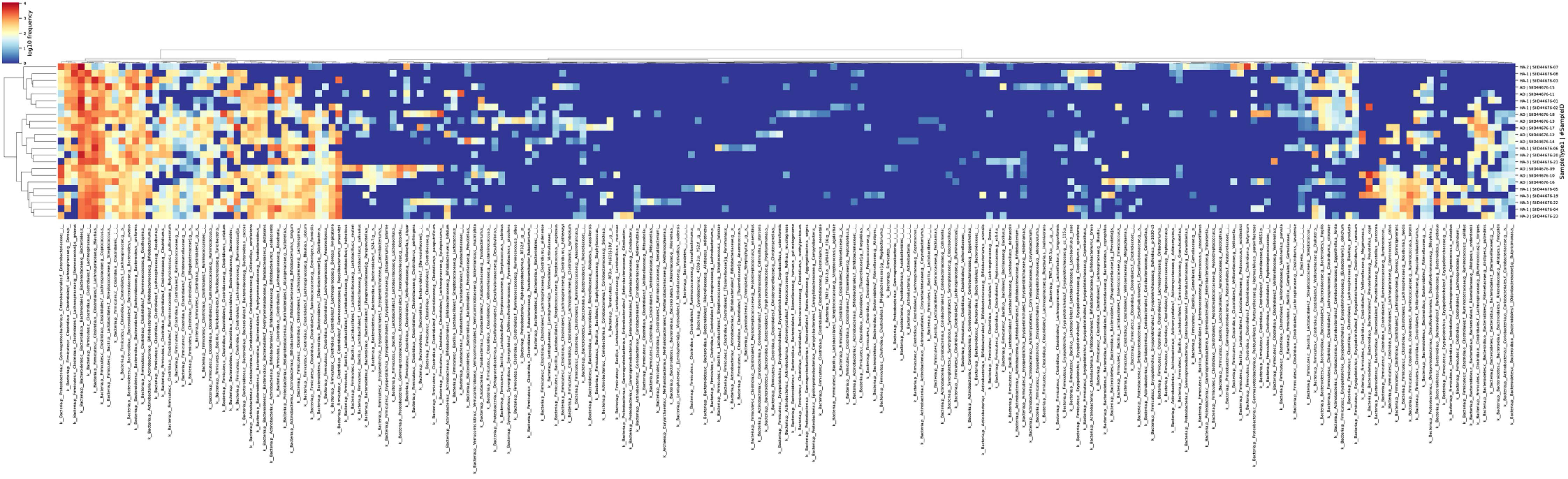

Supplement: Supplementary file 1 [file brainsci-16-00242-s001.zip › Fig S1.tif]

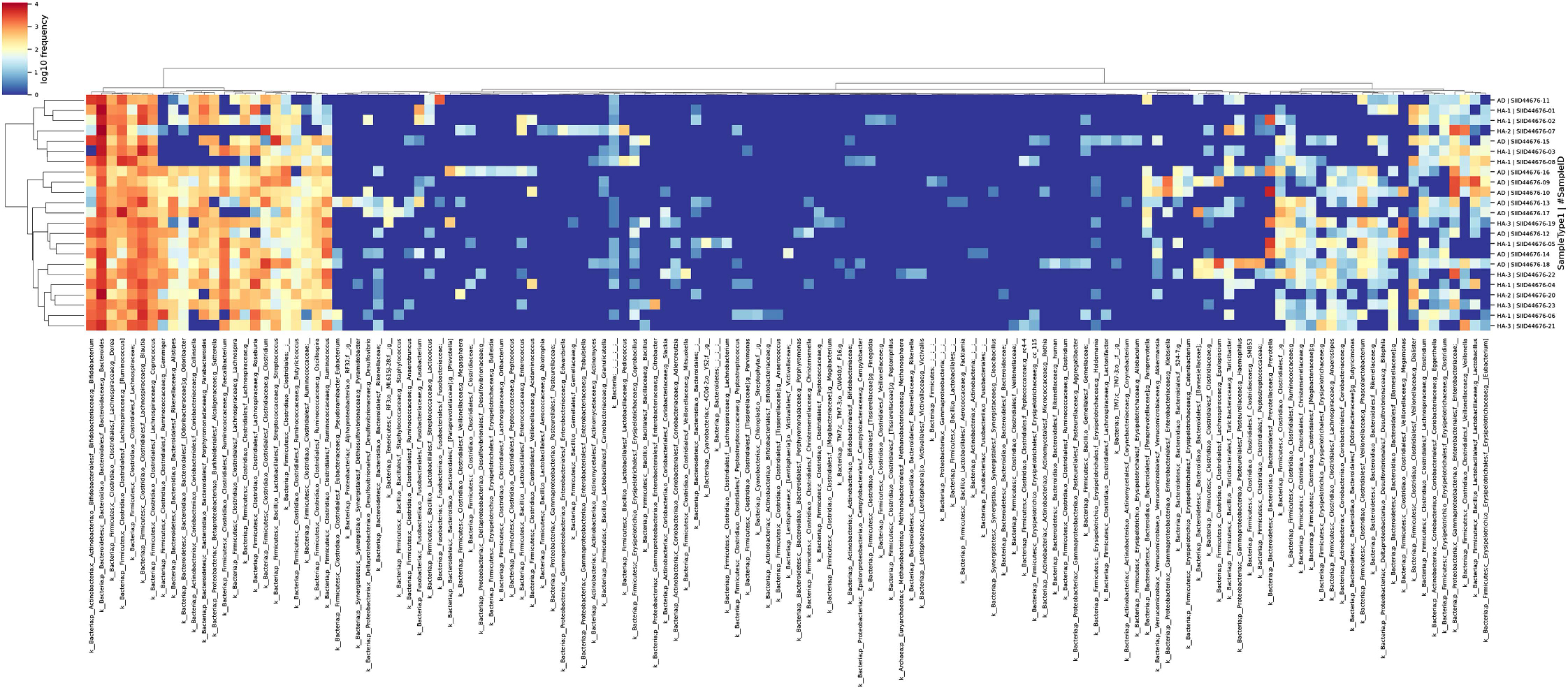

Supplement: Supplementary file 1 [file brainsci-16-00242-s001.zip › Fig S2.jpg]

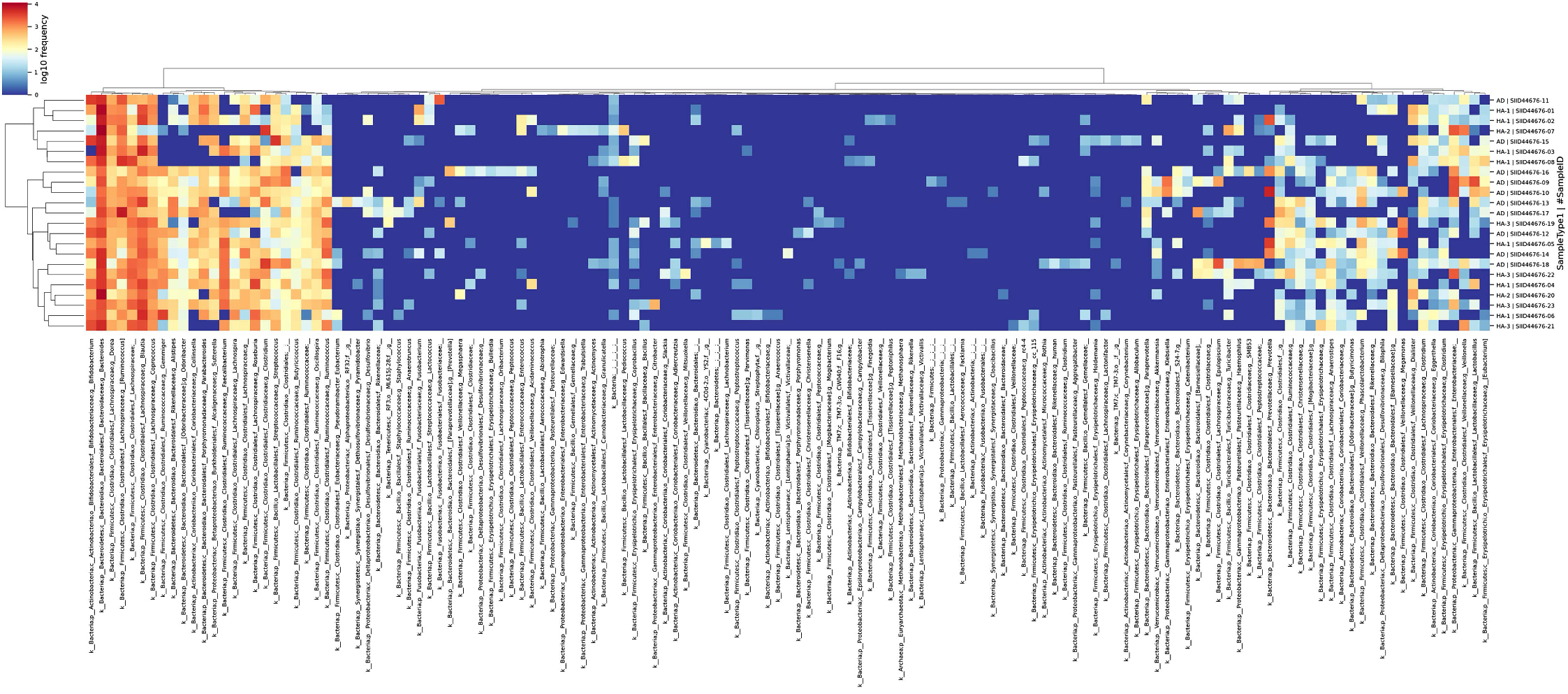

Supplement: Supplementary file 1 [file brainsci-16-00242-s001.zip › Fig S2.tif]

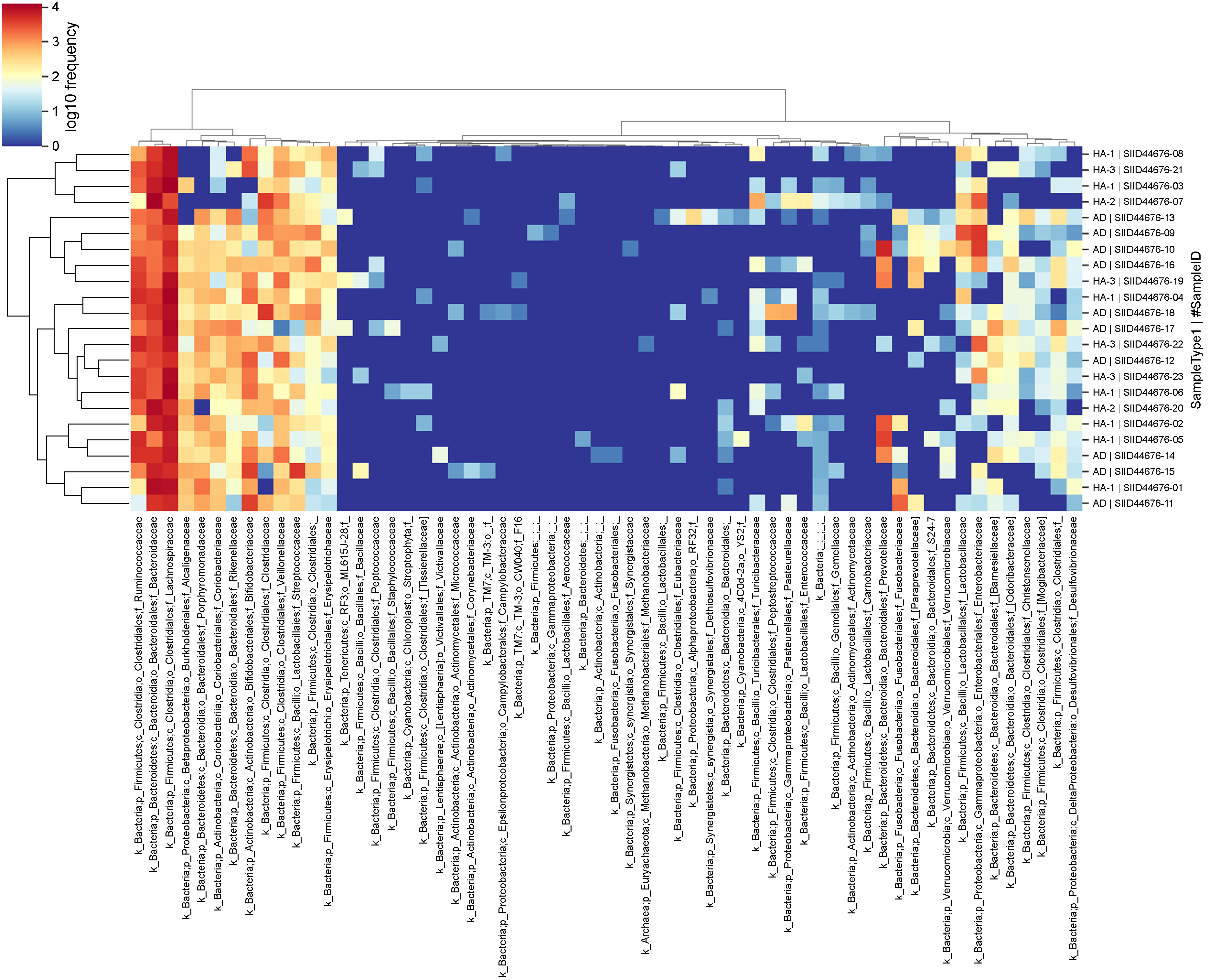

Supplement: Supplementary file 1 [file brainsci-16-00242-s001.zip › Fig S3.jpg]

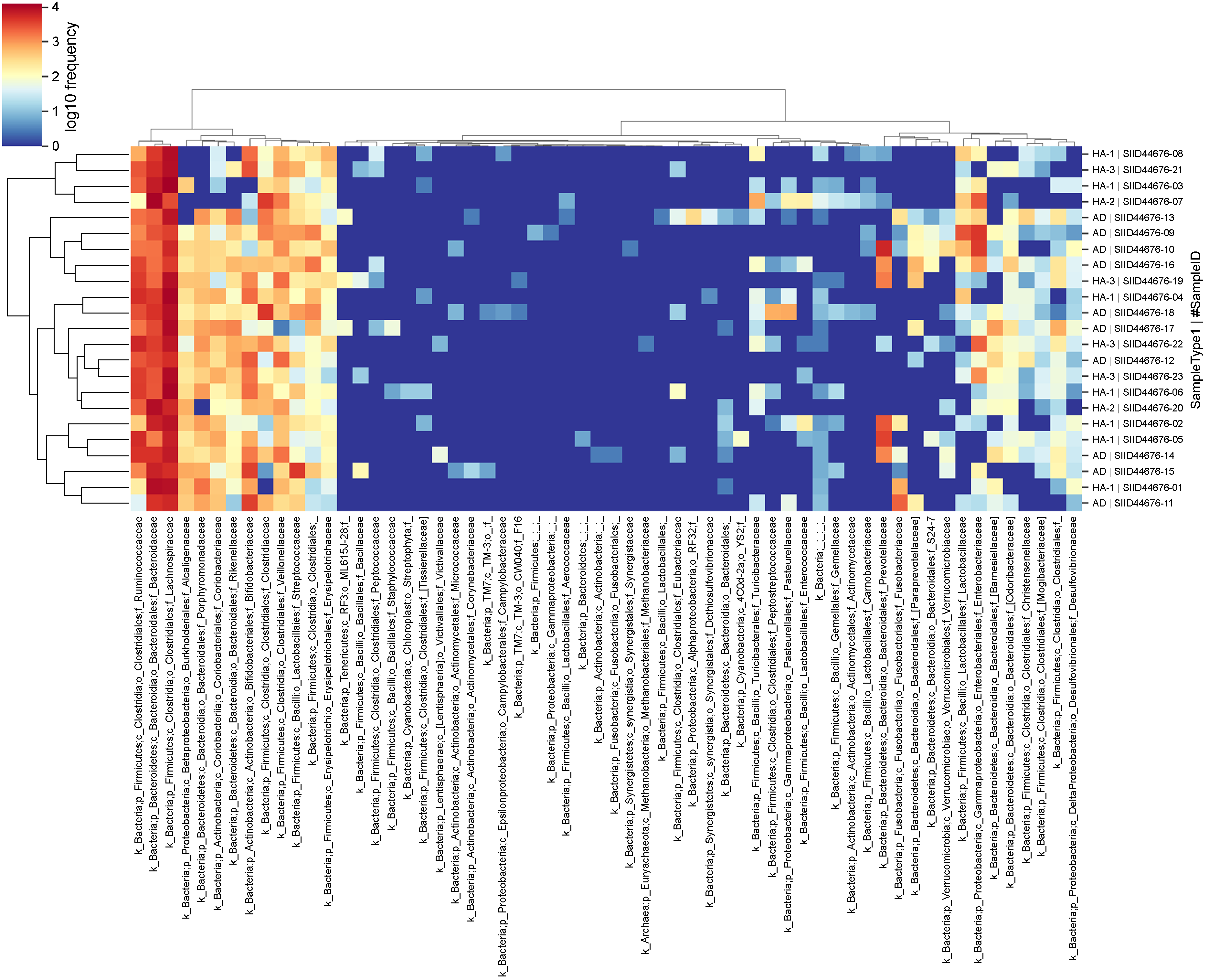

Supplement: Supplementary file 1 [file brainsci-16-00242-s001.zip › Fig S3.tif]

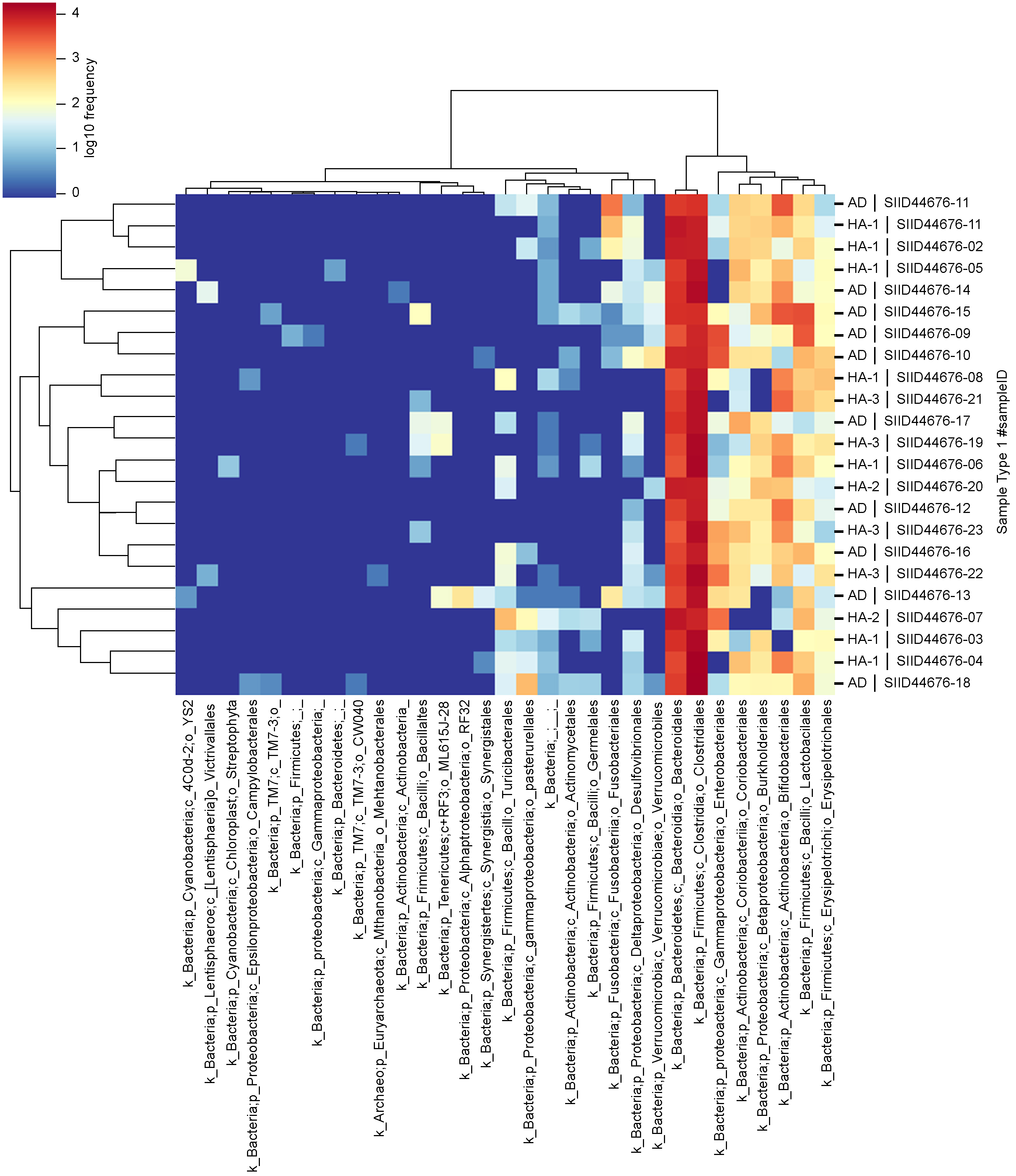

Supplement: Supplementary file 1 [file brainsci-16-00242-s001.zip › Fig S4.jpg]

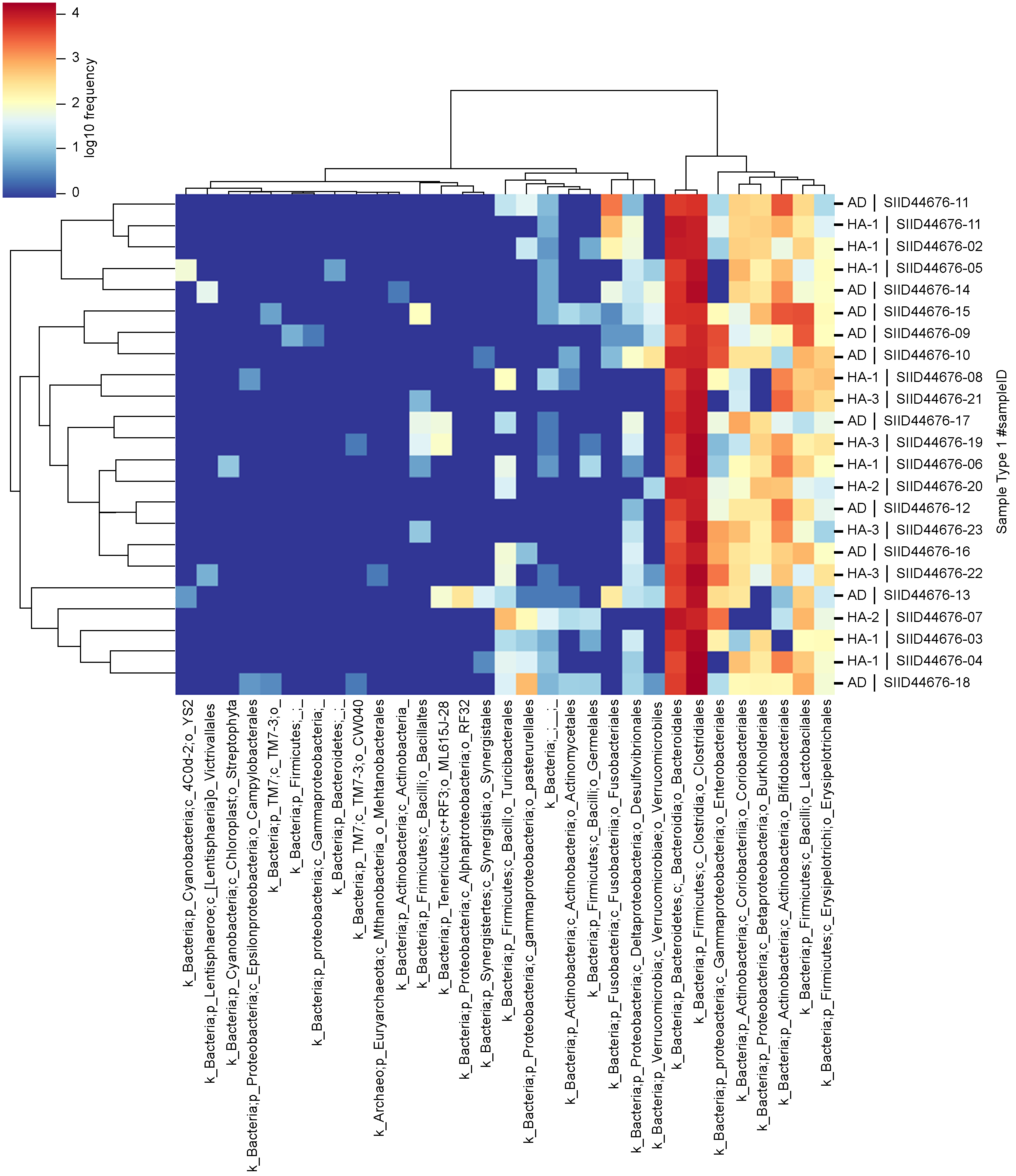

Supplement: Supplementary file 1 [file brainsci-16-00242-s001.zip › Fig S4.tif]

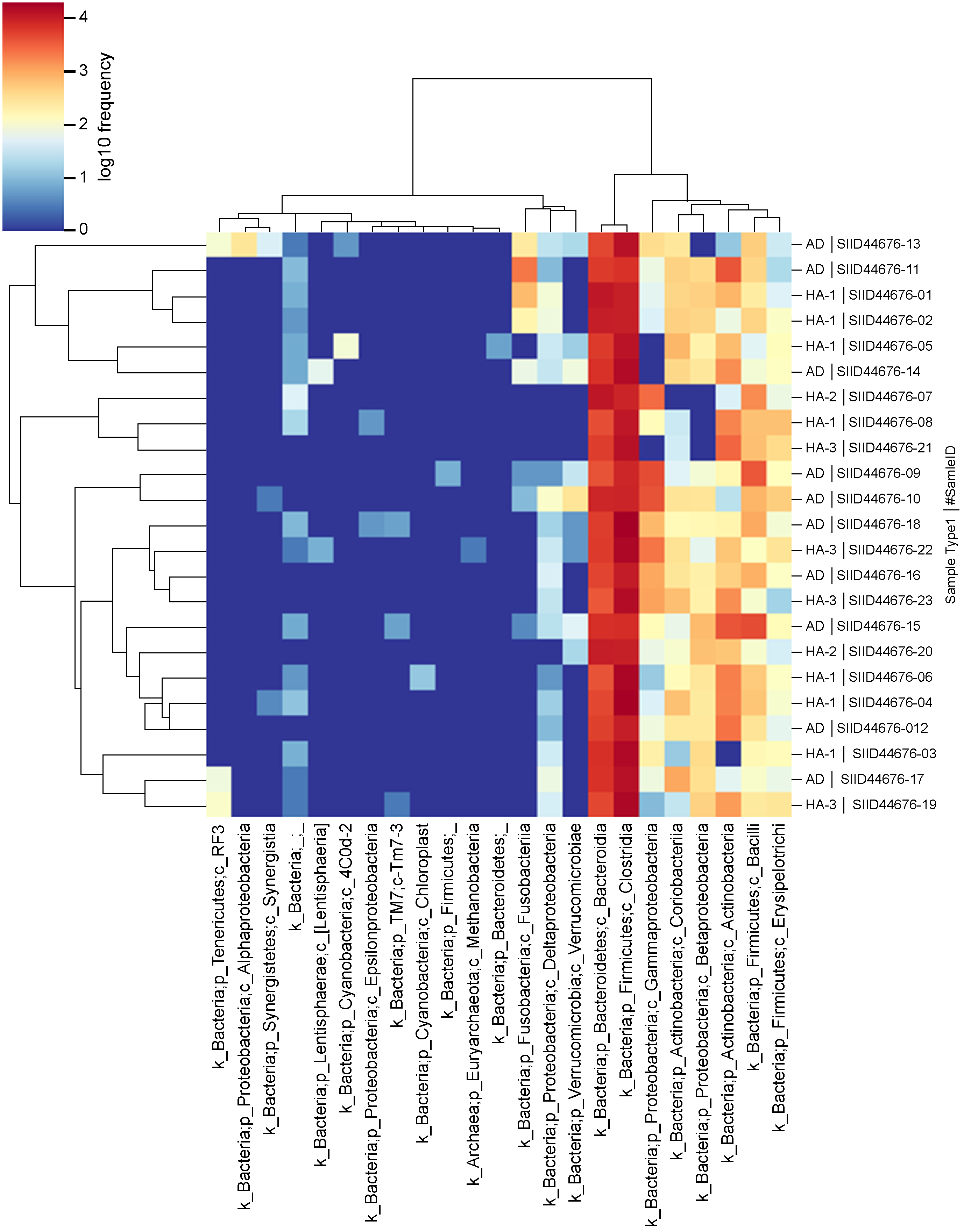

Supplement: Supplementary file 1 [file brainsci-16-00242-s001.zip › Fig S5.jpg]

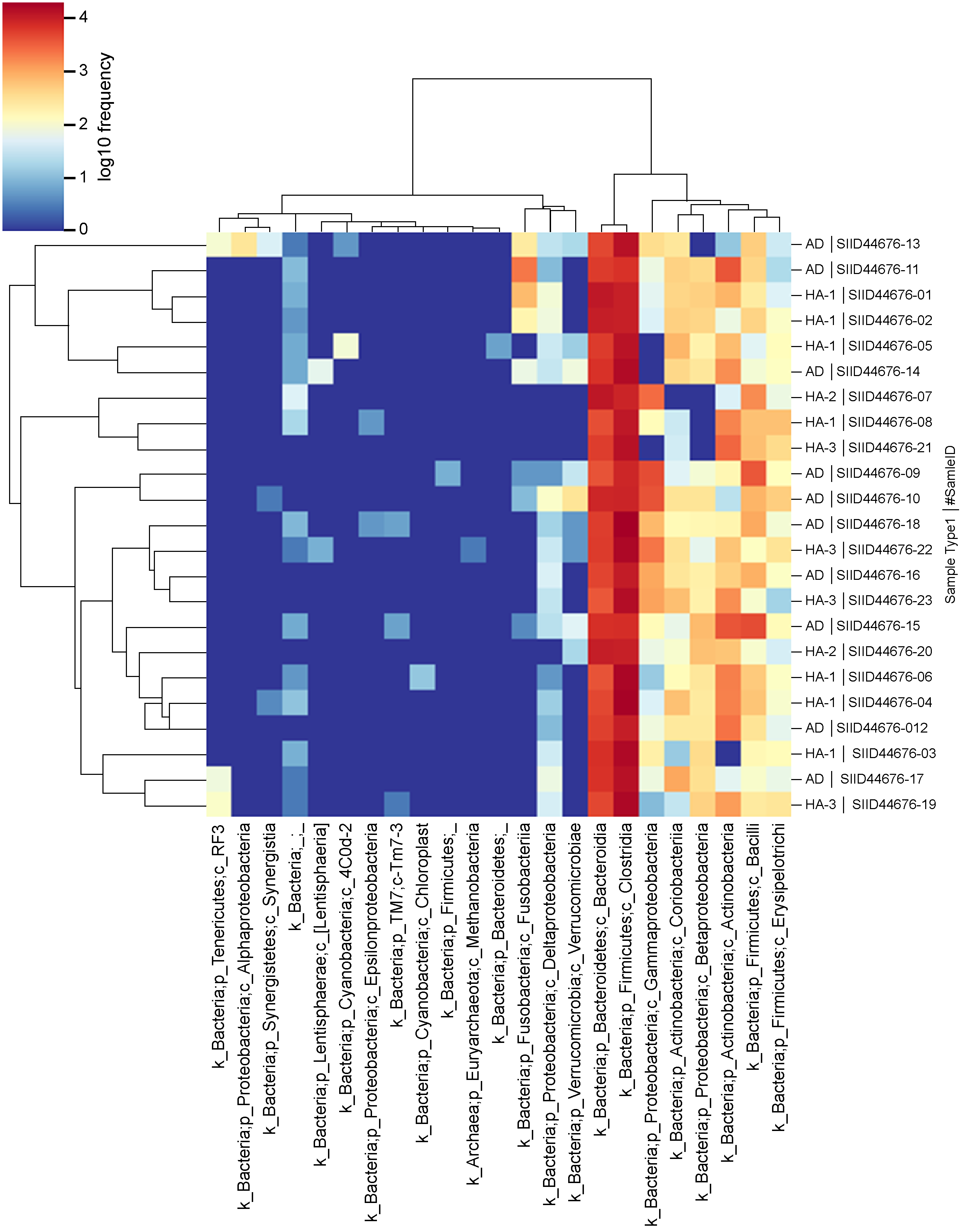

Supplement: Supplementary file 1 [file brainsci-16-00242-s001.zip › Fig S5.tif]

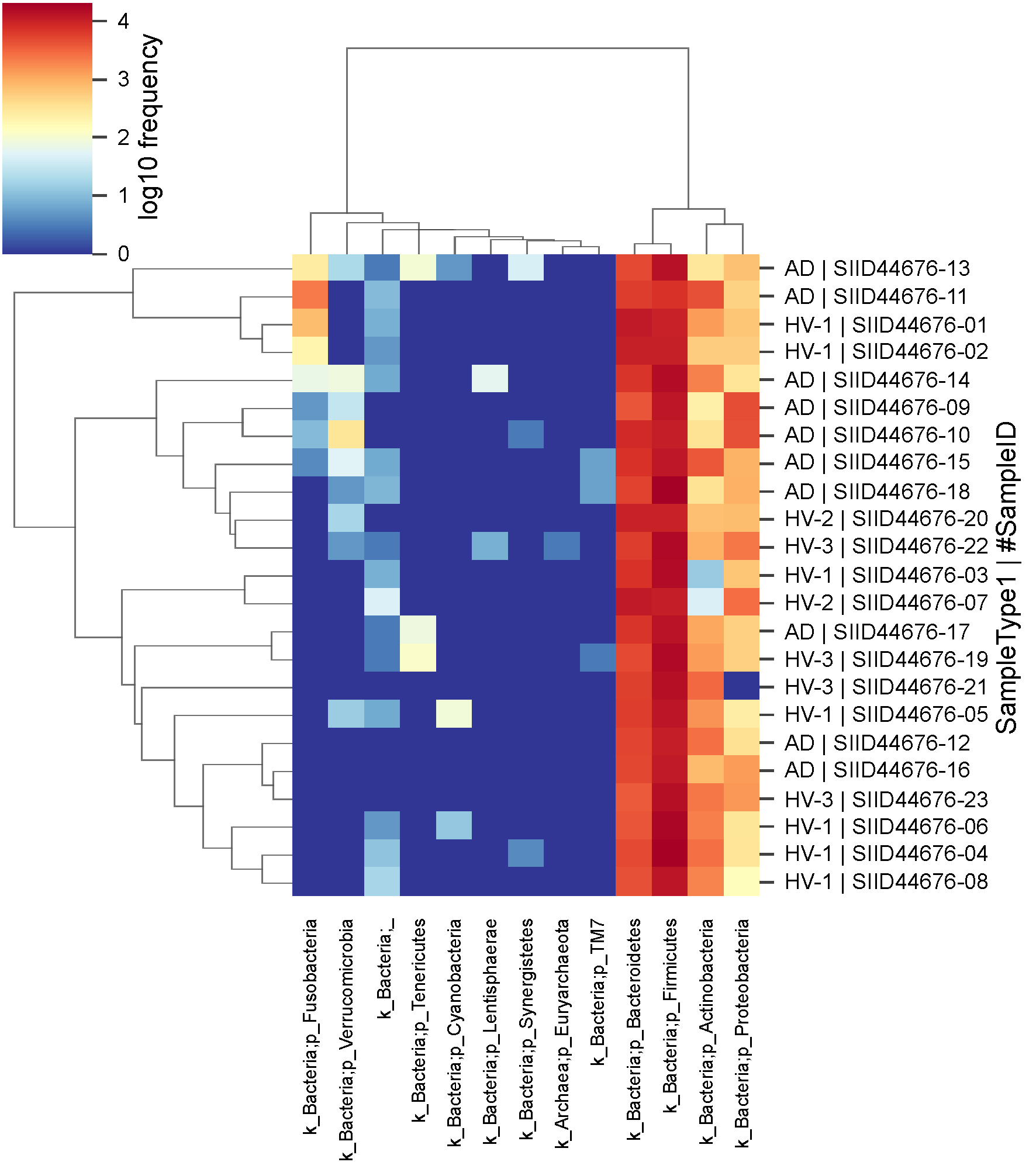

Supplement: Supplementary file 1 [file brainsci-16-00242-s001.zip › Fig S6.jpg]

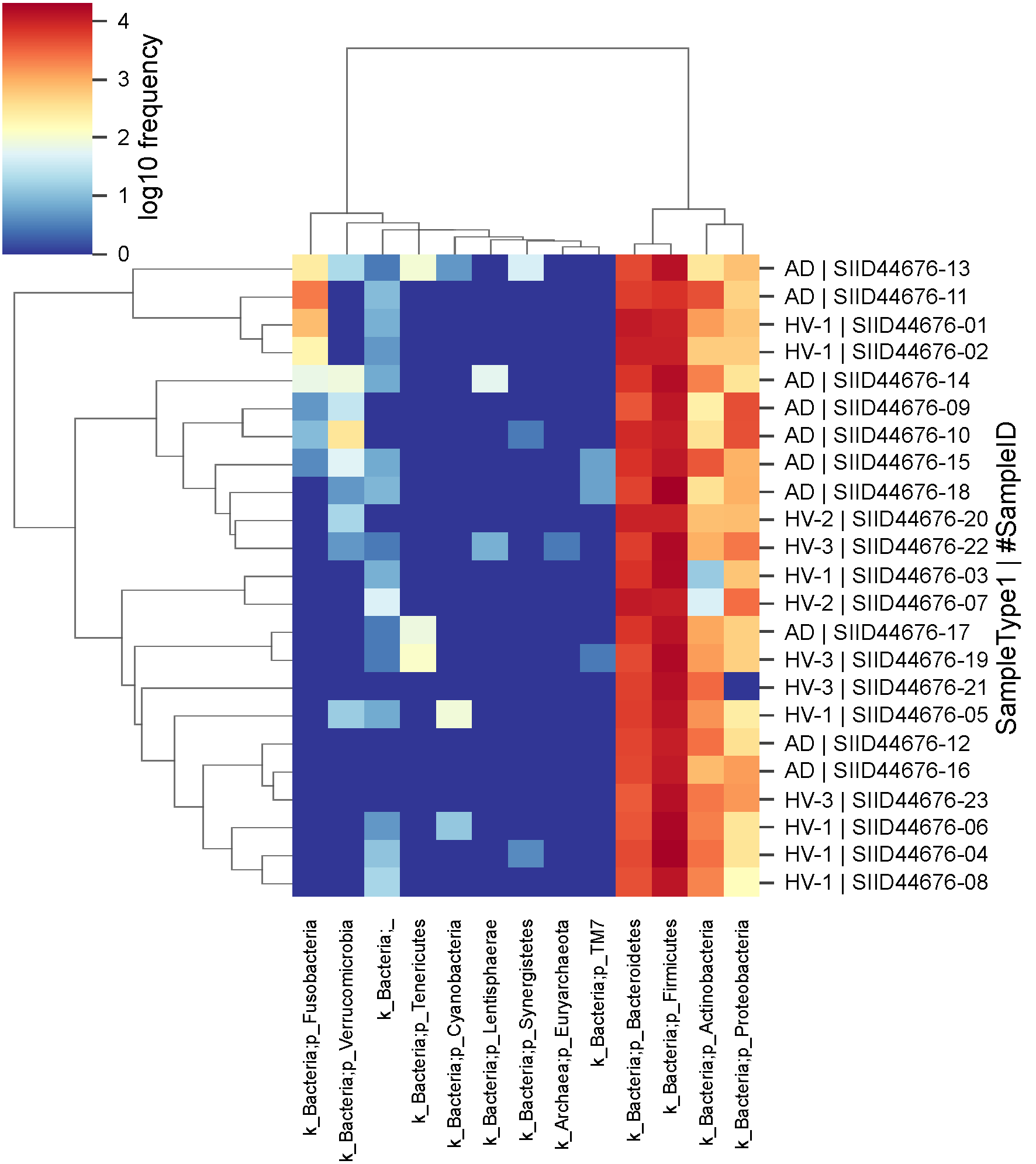

Supplement: Supplementary file 1 [file brainsci-16-00242-s001.zip › Fig S6.tif]

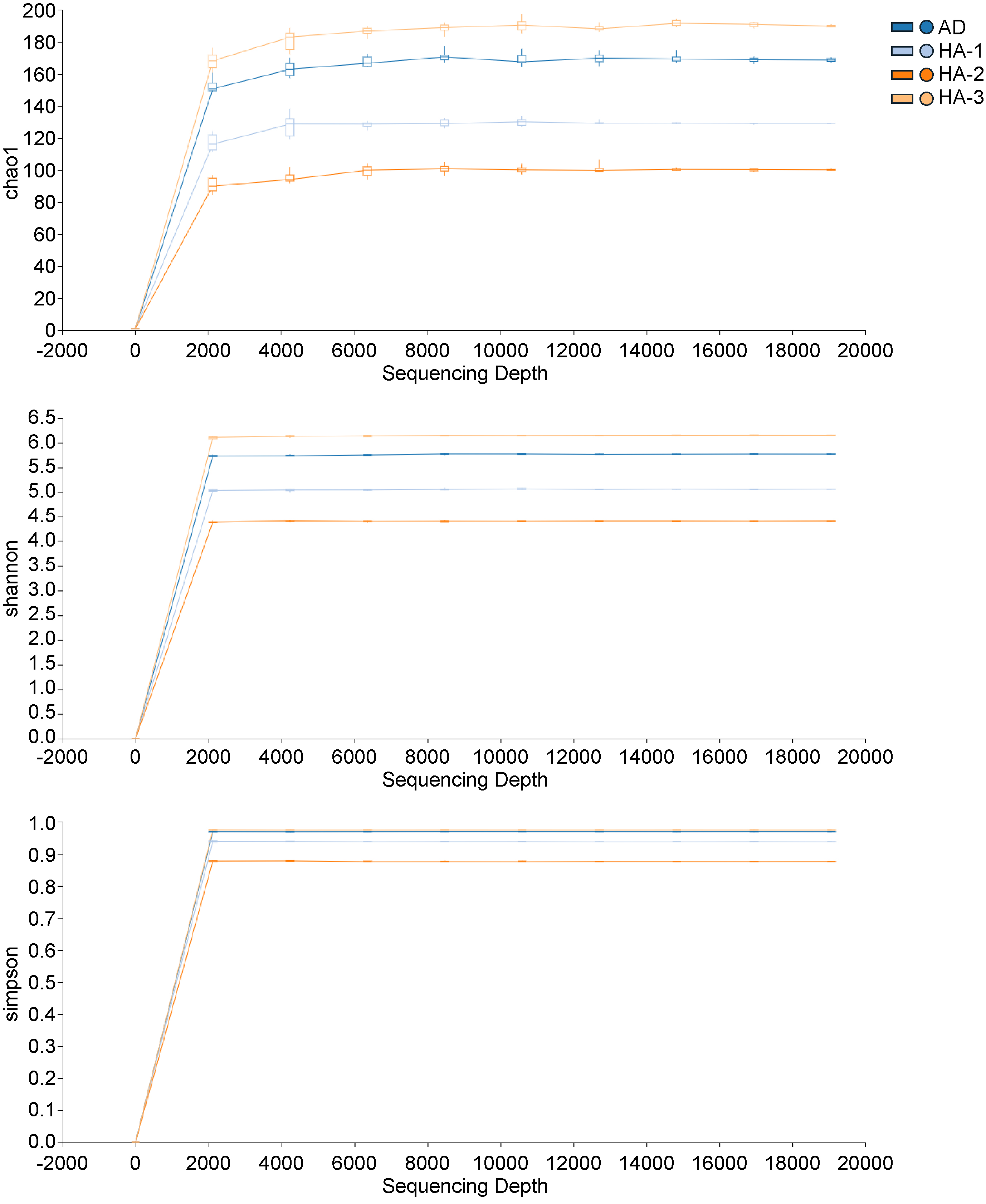

Supplement: Supplementary file 1 [file brainsci-16-00242-s001.zip › Fig S7.jpg]

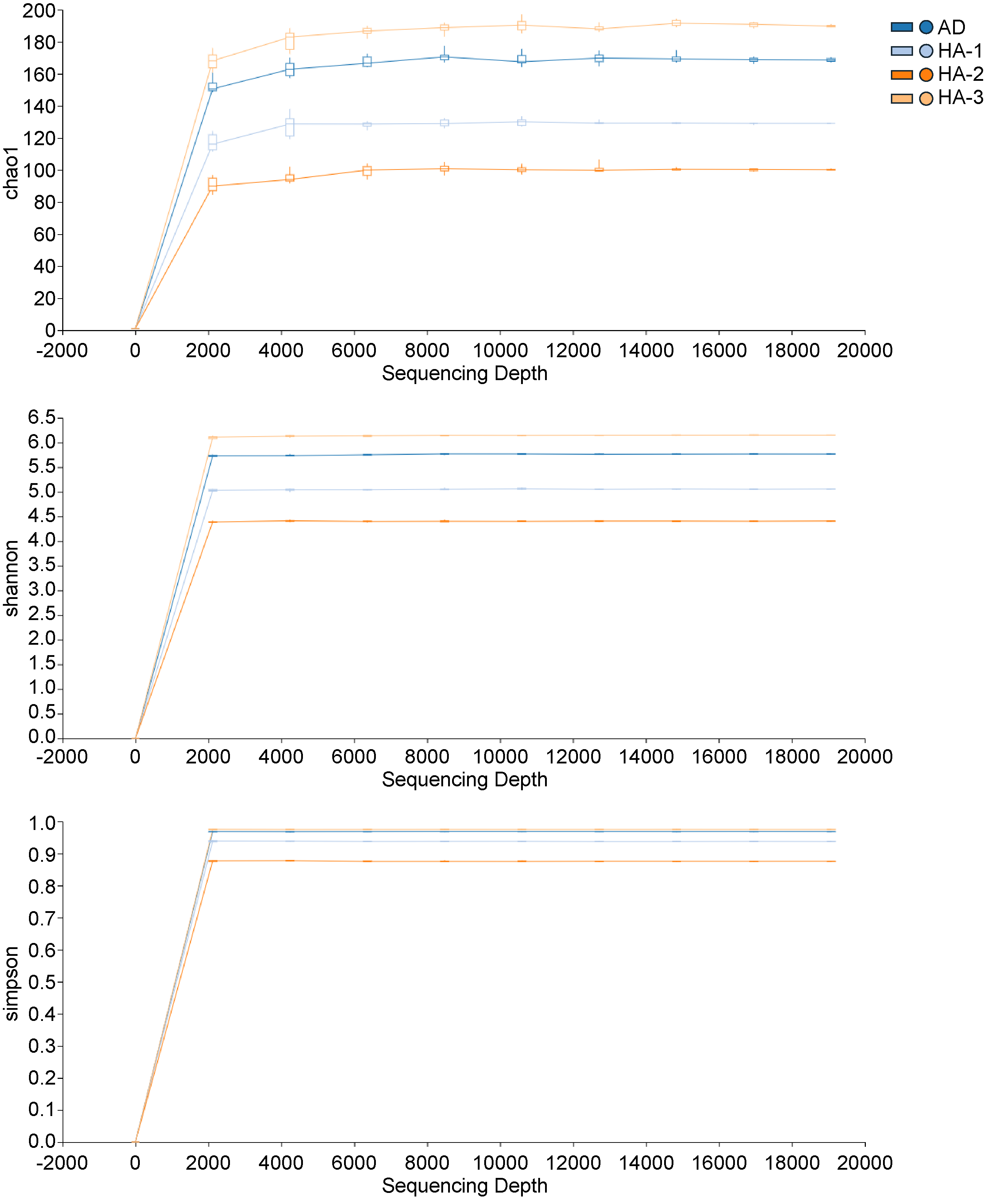

Supplement: Supplementary file 1 [file brainsci-16-00242-s001.zip › Fig S7.tif]

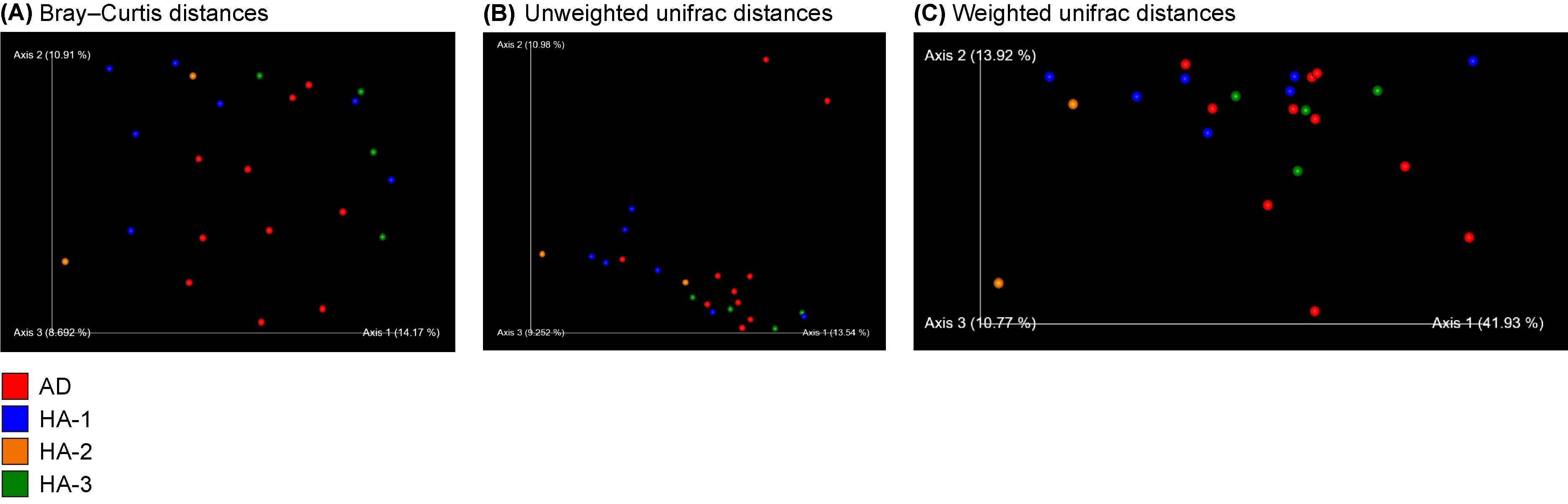

Supplement: Supplementary file 1 [file brainsci-16-00242-s001.zip › Fig S8.jpg]

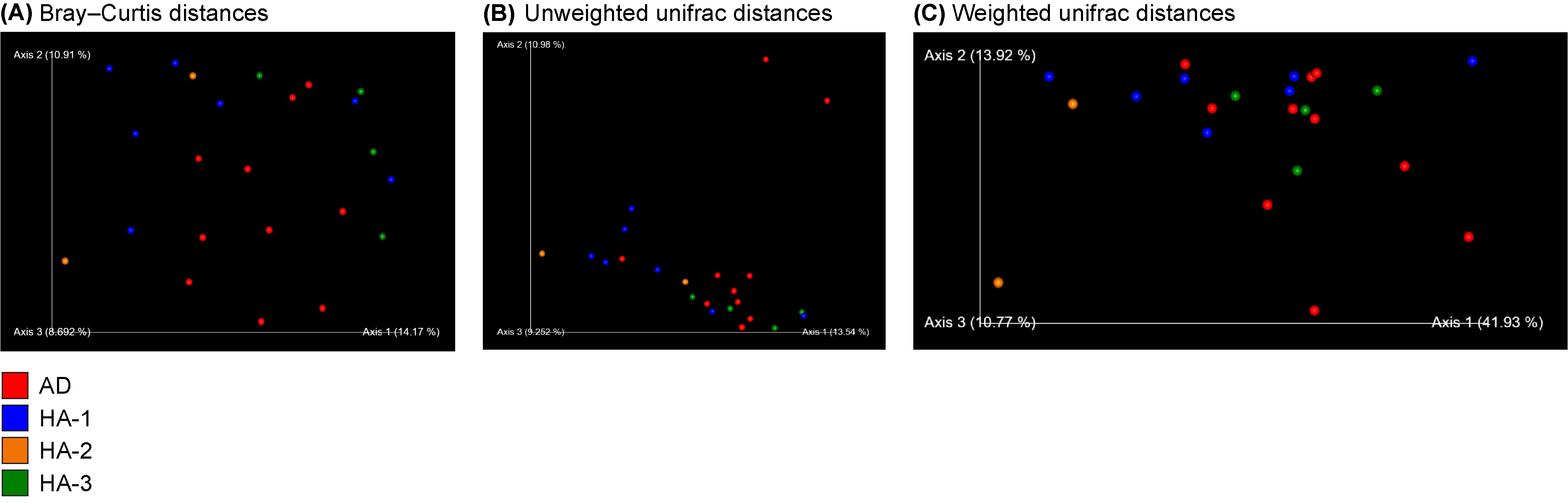

Supplement: Supplementary file 1 [file brainsci-16-00242-s001.zip › Fig S8.tif]
